# Revisiting CD8 ^+^ T Cells in Allergy: The Emerging Role of Tc2 Cells

**DOI:** 10.1111/sji.70146

**Published:** 2026-08-26

**Authors:** Sabrina de Souza Ferreira, Christopher Andrew Tibbitt

**Affiliations:** ^1^ Centre for Infectious Medicine, Department of Medicine Huddinge Karolinska Institutet and Karolinska University Hospital Stockholm Sweden; ^2^ Clinical Lung‐ and Allergy Research Unit, Department of Medicine Huddinge Karolinska Institutet Stockholm Sweden

## Abstract

Type 2 CD8^+^ T (Tc2) cells are increasingly recognised as contributors to allergic inflammation, yet remain less well understood than T helper 2 (Th2) cells and Group 2 innate lymphoid cells (ILC2s). Defined by production of IL‐4, IL‐5 and IL‐13, Tc2 cells have been implicated in eosinophilic inflammation, airway hyperresponsiveness, mucus production, and chronic tissue inflammation in diseases including asthma, atopic dermatitis, and chronic rhinosinusitis. Emerging evidence suggests that Tc2 differentiation and function are shaped by alarmins, lipid mediators, hypoxia, and immunometabolic pathways involving glycolysis, fatty acid metabolism, and serotonin signalling. Compared with Th2 cells, Tc2 cells may exhibit distinct features including tissue adaptation, responsiveness to innate inflammatory cues, and relative corticosteroid resistance, suggesting non‐redundant roles in sustaining chronic allergic inflammation. Current biologic therapies targeting Type 2 pathways likely influence Tc2 activity, although their effects on Tc2‐driven inflammation remain unclear. Here, we review current understanding of Tc2 cell phenotype, differentiation, immunometabolic regulation, and effector functions, and summarise evidence linking Tc2 cells to allergic disease. We also highlight key unanswered questions regarding Tc2 lineage stability, tissue residency, antigen specificity, and therapeutic targeting. Together, these findings position Tc2 cells as emerging amplifiers of Type 2 immunity and potential contributors to treatment‐resistant allergic disease.

## Graphical abstract

Tc2 CD8^+^ T cells in Type 2 inflammation: induction, tissue adaptation, metabolic programming, and therapeutic implications. Both naïve CD8^+^ T cells and pre‐committed Tc1 CD8^+^ T cells can be directed towards a Tc2 phenotype, characterised by secretion of the Type 2 cytokines IL‐4, IL‐5, IL‐9 and IL‐13, which in turn drive eosinophil recruitment, goblet cell hyperplasia and mucus production. Within the epithelial microenvironment, the local milieu shifts the balance from T1 towards T2 programming, and epithelial‐derived alarmins licence Tc2 cells for amplified cytokine output. This phenotype is supported by lipid‐dependent metabolic reprogramming, in which PPARγ acts as a transcriptional regulator promoting lipid uptake, cholesterol and lipid synthesis, and enhanced TCA cycle linked mitochondrial metabolism to sustain effector function. Clinically, Tc2 cells are relatively refractory to corticosteroids and thus contribute to steroid‐resistant Type 2 disease, whereas cytokine‐directed biologics can suppress their effector output; supporting Tc2 cells as both a therapeutic target and a candidate biomarker of treatment response.

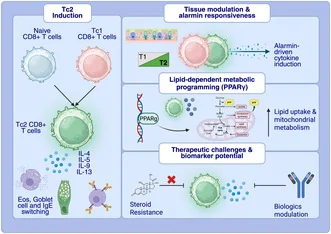

## 1 Introduction

Allergic diseases are increasing worldwide and represent a major and growing burden on global health systems [1]. Decades of research have established the central role of Type 2 lymphocytes, particularly T helper 2 (Th2) cells and Type 2 innate lymphoid cells (ILC2), in the initiation and maintenance of allergic inflammation [2]. Through the production of canonical cytokines, including interleukin (IL)‐4, IL‐5 and IL‐13, these cells orchestrate key features of Type 2 immunity, promoting immunoglobulin E (IgE) class switching, eosinophil recruitment and activation of tissue‐resident effector populations such as mast cells [3].

Despite the well‐defined roles of these cell types, the contribution of additional T‐cell subsets to allergic inflammation is increasingly recognised [4]. In contrast to their CD4^+^ counterparts, CD8^+^ T cells have traditionally been viewed as counter‐regulatory to Type 2 responses, largely due to their capacity to produce interferon‐γ (IFN‐γ) and support Type 1 immune responses [5]. However, emerging evidence indicates that CD8^+^ T cells display considerable functional plasticity within inflamed tissues [6, 7]. In particular, a subset of CD8^+^ T cells can adopt a Type 2 phenotype, characterised by expression of GATA3 and production of IL‐4, IL‐5 and IL‐13, and are now commonly referred to as Tc2 cells [8, 9, 10].

These observations suggest that Tc2 cells may contribute directly to the amplification and persistence of Type 2 inflammation, particularly at barrier sites such as the airway, skin and gastrointestinal tract [4, 11, 12]. Notably, in contrast to Th2 cells and similar to ILC2s, Tc2 cells appear relatively refractory to corticosteroids, raising the possibility that they may persist despite standard therapy and contribute to ongoing disease [13]. Recent studies have substantially expanded our understanding of Tc2 biology, identifying previously unrecognised mechanisms regulating their differentiation, metabolic programming, responsiveness to epithelial‐derived alarmins and persistence within inflamed tissues. Collectively, these findings suggest that Tc2 cells may represent a distinct component of Type 2 immunity with functions extending beyond those of a redundant source of Type 2 cytokines. In this review, we bring together these recent advances to provide an updated overview of Tc2 cell biology and its relevance to allergic disease.

## 2 Definition and Phenotype of Tc2 Cells

### 2.1 Cytokine Profile

Tc2 cells are defined functionally by their ability to produce canonical Type 2 cytokines, most notably interleukin (IL)‐4, IL‐5 and IL‐13 [4, 14]. These cytokines underpin key features of allergic inflammation, including immunoglobulin E (IgE) class switching, eosinophil recruitment and mucus production [4]. In this respect, Tc2 cells share substantial functional overlap with Th2 cells and ILC2 [4]. Furthermore, a subset of Tc2 cells has been shown to release IL‐9; a potent regulator of mast cell activation, mucus production and eotaxin release [15].

However, this cytokine profile contrasts sharply with that of classical Type 1 CD8^+^ T cells (Tc1), which are characterised by robust production of interferon‐γ (IFN‐γ) and tumour necrosis factor (TNF), alongside potent cytotoxic activity [16]. While Tc1 cells are specialised for the clearance of intracellular pathogens and tumour cells, Tc2 cells appear to be more closely aligned with immunomodulatory and tissue‐directed functions [17]. Notably, Tc2 cells exhibit reduced IFN‐γ production, although some degree of cytokine co‐expression has been reported, consistent with a degree of functional plasticity [18].

### 2.2 Surface Markers, Transcription Factors and Cytotoxic Features

The identification of Tc2 cells relies on a combination of functional and phenotypic characteristics, as no single surface marker uniquely defines this population (see Table 1). In humans, expression of CRTH2 (CD294), a receptor for prostaglandin D_2_, is widely used to enrich for Type 2‐polarised CD8^+^ T cells [19, 20]. Additional chemokine receptors, including CCR4 and CCR8, are frequently associated with Tc2 cells and are thought to facilitate migration to barrier tissues such as the skin and lung [21]. In contrast, expression of the Tc1 cell marker CXCR3 tends to be low or absent on Tc2 cells [26]. At the transcriptional level, Tc2 cells are characterised by high expression of GATA3, a key regulator of Type 2 lineage commitment. This contrasts with Tc1 cells, which are driven by transcription factors such as T‐bet that promote Type 1 cytokine production and cytotoxic differentiation [18]. Interestingly, despite reduced EOMES expression compared with Tc1 cells, human Tc2 cells maintain relatively high EOMES levels in relation to T‐bet [18].

**TABLE 1:** Key markers associated with Tc2 cells.

Category	Marker	Expression in Tc2 cells (relative to Tc1 cells)	Functional relevance	Reference(s)
Surface receptors	CRTH2 (CD294)	↑	PGD_2_ receptor; Tc2 identification, migration and activation	[18, 19]
	CCR4, CCR8	↑	Barrier tissue homing (skin and airway)	[20, 21]
	CysLT1R, BLT1	↑	Leukotriene responsiveness; inflammatory activation	[22, 23, 24, 25]
	CXCR3	↓	Reduced Type 1‐associated trafficking	[18, 26]
Transcription factors	GATA3	↑	Type 2 lineage commitment and cytokine production	[8]
	T‐bet	↓	Reduced Type 1 programme	[8, 18]
	HIF‐1α	↑	Enhances Tc2 responses under hypoxic conditions	[27]
	PPARγ	↑	Regulates lipid metabolism and Type 2 cytokine production	[18, 28]
Cytokines	IL‐4, IL‐5, IL‐13	↑	Canonical Type 2 effector cytokines	[9, 14]
	IL‐9	Variable ↑	Mast cell activation and mucosal inflammation	[15, 29]
Cytotoxic markers	Granzyme A/B, Perforin	↓	Reduced cytotoxicity relative to Tc1 cells	[30, 31, 32]
	Granzyme K	Variable ↑	Associated with chronic airway inflammation and remodelling	[33, 34]
Metabolic markers	GLUT1, CD36	↑	Enhanced glycolysis and fatty acid uptake	[18]
	MAOA	↑	Serotonin metabolism linked to Tc2 function	[18, 35, 36]
Tissue adaptation	CD69, CD103	Variable ↑	Putative tissue residency/TRM phenotype	[37, 38]

*Note:* Tc2 cells are characterised by expression of Type 2‐associated receptors, transcription factors and cytokines together with reduced expression of classical cytotoxic molecules relative to Tc1 cells. No single marker uniquely defines Tc2 cells, and identification typically relies on combined phenotypic and functional profiling.

Another defining feature distinguishing Tc2 from Tc1 cells is their relative cytotoxic capacity [30]. Classical Tc1 cells express high levels of Perforin, Granzymes A and B, and cytotoxic effector molecules, enabling efficient target cell killing [30, 31]. In contrast, Tc2 cells typically exhibit reduced expression of these cytolytic mediators and diminished killing capacity, suggesting a functional shift away from direct cytotoxicity towards cytokine‐mediated immunoregulation [32]. However, this distinction is not absolute. Under certain conditions, Tc2 cells may retain or reacquire elements of the cytotoxic programme, further supporting the concept that CD8^+^ T‐cell subsets exist along a spectrum of differentiation rather than as fixed lineages [6, 39].

## 3 Differentiation and Metabolic Regulation of Tc2 Cells

**FIGURE 1:**
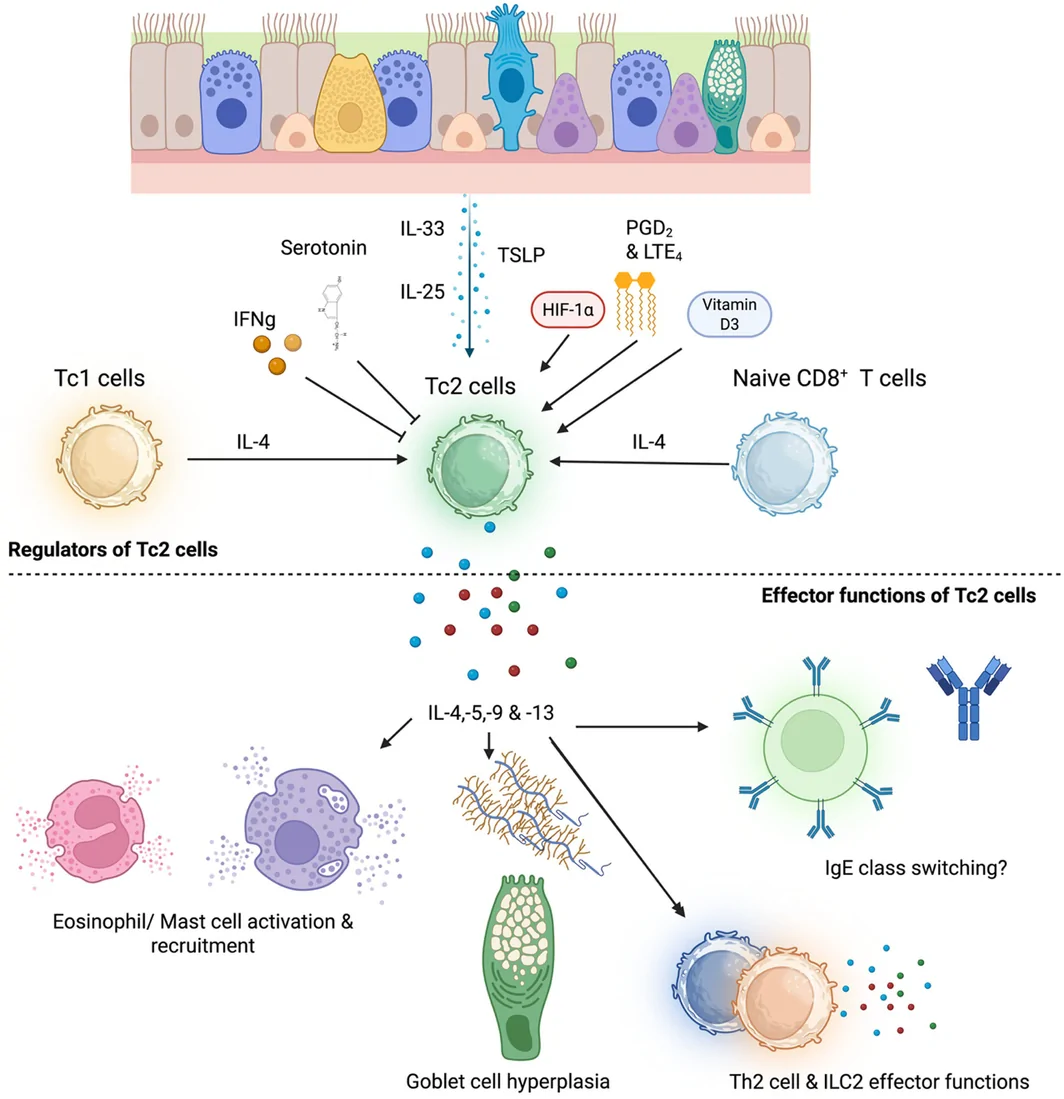
Differentiation cues and effector functions of Tc2 cells in Type 2 immunity. Tc2 differentiation may arise from naïve CD8^+^ T cells or through reprogramming of Tc1 cells under the influence of IL‐4, while epithelial‐derived alarmins (IL‐25, IL‐33 and TSLP), lipid mediators (PGD_2_ and LTE_4_), and environmental factors such as vitamin D₃ and hypoxia have been reported to support Tc2 development, activation and effector functions. Conversely, IFN‐γ, serotonin and other inflammatory cues may suppress Tc2 differentiation. Activated Tc2 cells produce Type 2 cytokines including IL‐4, IL‐5, IL‐9 and IL‐13, contributing to eosinophil and mast cell recruitment/activation, goblet cell hyperplasia and mucus production, and amplification of Type 2 immune responses through interactions with Th2 cells and group 2 innate lymphoid cells (ILC2s). Potential roles for Tc2 cells in promoting B cell activation and IgE class switching remain incompletely defined and require further investigation.

In vitro studies have demonstrated that exposure of naïve CD8^+^ T cells to interleukin (IL)‐4 during activation is sufficient to induce a Type 2 phenotype including the acquisition of Type 2 cytokine production, including IL‐4, IL‐5 and IL‐13, alongside upregulation of the lineage‐defining transcription factor GATA3 (summarised in Figure 1) [9]. These findings parallel differentiation pathways described for CD4^+^ T helper subsets and support the concept that CD8^+^ T cells retain substantial functional plasticity in response to their cytokine environment [4]. However, the stability of the Tc2 phenotype remains incompletely defined. Some studies indicate that Tc2 cells can retain their Type 2 characteristics upon antigen re‐encounter, suggesting a degree of lineage commitment [40]. In contrast, other reports suggest that sustained or repeated exposure to Type 2‐polarising conditions is required to maintain this phenotype, with cells otherwise reverting towards a Type 1 programme, including reacquisition of IFN‐γ production and cytotoxic function [41, 42]. These discrepancies likely reflect differences in experimental systems, strength and duration of stimulation, and the presence or absence of reinforcing environmental cues.

Importantly, at barrier tissues, epithelial‐derived cytokines such as IL‐25, IL‐33 and TSLP, so‐called ‘Alarmins’, play key roles in shaping CD8^+^ T‐cell fate, with IL‐33 being shown to play an especially potent role in induction of the Tc2 effector functions in both mice and humans potentially by indirectly inducing IL‐4 release from other cells within the tissue microenvironment as well as the suppression of IFNγ release from CD8^+^ T cells [10, 13, 18]. Moreover, studies in HDM challenged IL‐33 ‘knockout’ mice demonstrated a selective loss of IL‐5‐producing Tc2 cells, whereas IL‐5 production by Th2 cells was largely preserved, highlighting a non‐redundant role for IL‐33 in Tc2 responses [13]. Interestingly, IL‐33 deficiency also reduced IL‐9 production while largely preserving IL‐4 expression, suggesting that IL‐33 differentially regulates the Type 2 cytokine programme [13]. Consistent with these findings, IL‐33 potently induces IL‐5 production by human Tc2 cells, whereas maximal IL‐13 production requires the combined activity of IL‐33, TSLP and IL‐25, illustrating the cooperative role of epithelial alarmins in regulating distinct Tc2 effector function [18]. Together, these findings highlight that Tc2 differentiation is a dynamic and context‐dependent process, shaped by both cytokine milieu and tissue‐specific signals. These studies shift the prevailing model of Tc2 differentiation away from one driven solely by antigen and IL‐4 towards one in which epithelial‐derived danger signals actively shape Tc2 effector programming within inflamed tissues. Whether Tc2 cells establish long‐lived tissue‐resident memory populations remains unclear. Emerging evidence from allergic airway models and parallels with Th2 Tissue Resident Memory (TRM) biology suggest that local tissue cues, including IL‐33, may promote resident‐like adaptation of Tc2 cells, although definitive lineage and persistence studies are lacking [13, 43].

Among the non‐cytokine pathways regulating Tc2 biology, lipid mediators appear to play a particularly important role. Tc2 cells express high levels of CRTH2, the receptor for prostaglandin D_2_ (PGD_2_), together with leukotriene receptors including CysLT1 and, in some contexts, BLT1 [22, 23, 24]. Through these receptors, Tc2 cells can respond directly to eicosanoids in a manner that is independent of T‐cell receptor stimulation. Experimental studies have demonstrated that PGD_2_ and LTE_4_ act synergistically to promote Tc2 recruitment, activation and cytokine production, including IL‐5 and IL‐13 release [19]. This pathway may be especially relevant within allergic tissues, where mast cell activation following IgE crosslinking provides a local source of lipid mediators capable of sustaining Tc2 responses [23]. In turn, Tc2‐derived cytokines promote eosinophil recruitment and survival, establishing a feed‐forward inflammatory circuit linking mast cells, eosinophils, structural cells and Type 2 lymphocytes.

The inflammatory tissue microenvironment appears central to the regulation of Tc2 effector function. In addition to cytokine and alarmin exposure, recent evidence suggests that Tc2 cells undergo substantial metabolic reprogramming that supports their inflammatory activity. Human Tc2 cells exhibit increased glycolytic activity, enhanced mitochondrial respiration and elevated fatty acid uptake relative to non‐Type 2 CD8^+^ T‐cell populations [18, 44]. These cells also display increased expression of metabolic regulators including GLUT1 and CD36, consistent with a metabolically active effector phenotype adapted to inflamed tissues [18]. Functional studies demonstrate that Tc2 cytokine production depends on coordinated glycolytic and lipid metabolic pathways. Inhibition of glycolysis, fatty acid oxidation or PPARγ signalling significantly impairs production of IL‐4, IL‐5 and IL‐13, indicating that Tc2 effector function is tightly coupled to cellular metabolic state [18]. Recent work from our group further suggests that serotonin signalling may contribute to the regulation of Tc2 effector function. Transcriptomic analysis identified elevated expression of MAOA and other serotonin metabolism‐associated pathways in Tc2 cells, distinguishing them from Tc1 populations [18]. Pharmacological inhibition of MAOA significantly reduced IL‐4, IL‐5 and IL‐13 production without altering IFN‐γ expression, indicating that serotonin catabolism selectively supports Type 2 cytokine production in Tc2 cells and in contrast to Tc1 cells [35, 36]. Consistent with these findings, individuals exposed to Selective Serotonin Reuptake Inhibitors (SSRIs) exhibited reduced frequencies of IL‐5^+^ and IL‐13^+^ Tc2 cells alongside increased IFN‐γ–producing Tc2 cells following ex vivo stimulation, supporting a link between serotonin signalling, metabolic state and Tc2 functional plasticity in humans [18]. Murine studies have also shown that hypoxic conditions also support enhanced Tc2 cell effector functions mediated by HIF‐1α [27]. Vitamin D3 is another regulator of Tc2 cells, promoting IL‐13 expression while lowering IFN‐γ levels in part via regulation of the steroidogenic enzyme, CYP11A1 [42, 45]. Together these findings show how the local microenvironment shapes Tc2 cell activity in allergic inflammation.

Changes in the frequency of innate‐like CD8^+^ T cells have also been observed cells in overweight and obese individuals with asthma suggesting that host metabolic status can influence CD8 T‐cells frequencies in vivo [46]. Taken together, these studies point towards a core immunometabolic profile being shared across Type 2 lymphocyte subsets in allergic responses [28, 47, 48, 49]. In chronically inflamed tissues such as the asthmatic airway, these environmental cues may alter Tc2 lineage stability and sustain inflammatory cytokine production independently of continuous antigen stimulation.

## 4 Effector Functions in Allergic Inflammation

Tc2 cells contribute to allergic inflammation primarily through the production of canonical Type 2 cytokines, notably interleukin (IL)‐4, IL‐5, IL‐9 and IL‐13 (see Figure 1). These cytokines mediate key effector pathways that underpin the development and persistence of allergic disease. IL‐5 plays a central role in the recruitment, activation and survival of eosinophils, thereby promoting eosinophilic inflammation within affected tissues [2]. Through the production of IL‐4, Tc2 cells can also contribute to B cell class switching to immunoglobulin E (IgE), a defining feature of allergic sensitization although evidence in vivo of Tc2‐B cell interactions remains scarce [22]. It could be hypothesised that while CD4^+^ T cells remain the dominant providers of B cell help, Tc2 cells may provide additional or localised support within inflamed tissues. In addition, IL‐13 drives mucus hypersecretion, airway hyperresponsiveness and epithelial cell activation, particularly within the respiratory tract. Collectively, these cytokine‐mediated effects position Tc2 cells as active contributors to the amplification of Type 2 immune responses at barrier sites. In addition to these canonical mediators, Tc2 cells have also been reported to produce IL‐9 in certain contexts [13, 50]. Although not a defining feature of all Tc2 cells, IL‐9 may further amplify allergic inflammation through its effects on mast cell activation and mucosal responses [29]. There is also transcriptional evidence of IL‐31 expression in activated human Tc2 cells [18], suggesting possible neural crosstalk given its role in the ‘itch’ response [51].

Mast cells are another key interacting partner. Both Tc2 cells and mast cells respond to lipid mediators such as prostaglandin D_2_ via receptors including CRTH2, enabling coordinated recruitment and activation within inflamed tissues [23, 25]. Tc2‐derived cytokines, including IL‐4, IL‐13 and potentially IL‐9, may further enhance mast cell activation, survival and mediator release. Conversely, mast cell‐derived products, including histamine, serotonin, cytokines and lipid mediators, can influence Tc2 cell function and migration, contributing to the regulation and amplification of local Type 2 responses [12, 18, 52].

Interactions between Tc2 cells and ILC2 are likely to be particularly important at barrier sites. Both populations produce overlapping sets of Type 2 cytokines and respond to epithelial‐derived alarmins such as IL‐25, IL‐33 and thymic stromal lymphopoietin [13, 53]. This shared responsiveness may result in synergistic amplification of cytokine production within tissues. In addition, ILC2‐derived cytokines may help sustain Tc2 function in a T‐cell receptor‐independent manner, although the extent and directionality of these interactions remain to be fully defined. Beyond these interactions, Tc2 cells are likely to engage with antigen‐presenting cells, including dendritic cells and macrophages, which shape their activation and differentiation through cytokine and co‐stimulatory signals. Reciprocally, Tc2‐derived cytokines may influence the function and polarisation of these cells, further reinforcing a Type 2‐skewed microenvironment. Collectively, these interactions position Tc2 cells as integral components of a broader cellular network that coordinates allergic inflammation. Rather than acting as isolated effector cells, Tc2 cells participate in dynamic and context‐dependent crosstalk with multiple immune populations, contributing to both the amplification and persistence of Type 2 immune responses under the regulation of shared transcriptional programmes regulated primarily by GATA3 [54].

## 5 Tc2 Cells in Human Allergic Diseases

### 5.1 Asthma and Chronic Rhinosinusitis (CRS)

Removal of CD8^+^ T cells by antibody mediated depletion or by genetic tools in allergic models of airway inflammation ablates the Type 2 inflammatory response suggestive of an important role for Tc2 cells in the airways [55, 56, 57]. Evidence supporting a pathogenic role for Tc2 cells in human disease has been most widely studied in eosinophilic asthma [4]. Studies of bronchoalveolar lavage (BAL) fluid and bronchial biopsies have demonstrated enrichment of CD8^+^ T cells within asthmatic airways, with several reports identifying stronger associations between Tc2 cell frequencies and airway hyperresponsiveness than for CD4^+^ T cells [13, 37, 58, 59, 60]. Indeed, in two independent cohorts of severe eosinophilic asthma, only Tc2 cells were found to be enriched in the blood and airways of such individuals in contrast to Th2 cells where no significant change in frequency was found [19]. A similar observation has also been made in non‐IgE mediated eosinophilia where significantly higher frequencies of IL‐5^+^ and IL‐3^+^ Tc2 cells were found in circulation of patients [61]. Post‐mortem studies in fatal asthma provide additional support for a pathogenic role of CD8^+^ T cells [62]. In these settings, CD8^+^ T cells may outnumber CD4^+^ T cells within airway tissues and display evidence of activation together with increased expression of cytotoxic mediators such as Perforin and Granzyme B (GZMB) [63]. Notably, fatal asthma has also been associated with an increased ratio of Type 2 cytokine‐producing relative to Type 1 cytokine‐producing CD8^+^ T cells, implicating Tc2‐skewed responses in severe airway pathology [64, 65].

Collectively, these findings suggest that Tc2 cells are not merely bystanders within allergic inflammation but may represent important effector populations contributing to airway dysfunction and disease severity [66]. Longitudinal studies further support a relationship between CD8^+^ T cells and progressive airway disease [67]. In cohorts of patients with asthma followed over extended periods, increased bronchial CD8^+^ T‐cell numbers have been associated with greater decline in lung function over time, including reductions in post‐bronchodilator FEV1 [33]. Notably, again, these associations appear stronger for CD8^+^ than CD4^+^ T‐cell populations. These observations raise the possibility that persistent Tc2 or Tc2‐like CD8^+^ T‐cell responses may contribute not only to inflammatory activity but also potentially to chronic airway remodelling and disease progression via mediators such as Granzyme K (GZMK) as found in chronic rhinosinusitis (CRS) [34, 68]. It is thought that GZMK plays an important role in cleaving complement components, including C2–C5, to sustain inflammation as well as a role in organising Tertiary Lymphoid Structures (TLS). Interestingly, tissue expression of GZMK is a better predictor of disease severity than either eosinophilia or tissue IL‐5 expression [34]. Single cell transcriptomic analysis has also that GZMK^+^ Tc cells interact with fibroblasts via the CXCR4‐CXCL12 axis and, in turn, can induce neutrophil infiltration [68]. How these cells relate to Tc2 cells remains unclear as some studies show that nasal polyp CD8^+^ T cells have a reduction in the expression of cytotoxic mediator GZMB and elevated cytokine expression, including Type 2 cytokines, compared to the blood counterparts [69, 70, 71], although others have observed increased expression of IFN‐γ with an enrichment for Epstein–Barr Virus (EBV)–specific TCRs within this subset [34, 68]. These observations raise the intriguing possibility that Tc2 cells represent one component of a broader spectrum of pathogenic CD8^+^ T‐cell states that emerge within chronically inflamed barrier tissues rather than a single discrete lineage.

Respiratory viral infections represent a major trigger of asthma exacerbations and may play an important role in shaping Tc2 responses within the airway [10]. Experimental studies have demonstrated that virus‐specific CD8^+^ T cells can acquire Type 2 cytokine‐producing capacity as a result of viral induced alarmin release, resulting in increased IL‐5 production and airway eosinophilia. Respiratory syncytial virus (RSV) infection has been particularly implicated in this process, with early‐life RSV exposure associated with enhanced Tc2 responses, mucus production, and airway hyperresponsiveness upon reinfection in murine models [72, 73, 74]. Similarly, Rhinovirus infection has been shown to induce IL‐4 expression in CD8^+^ T cells with impaired Type 1 responses in children with wheezing [75]. However, in a more recent study, there was significant heterogeneity in CD8^+^ T‐cell compartment with a complex mixture of both IFN‐γ and IL‐17a Tc cell effectors present although these populations also expressed PD‐1 and ICOS that can be enriched on Type 2 cells such as ILC2 [76, 77, 78]. Taken together, these findings support a model in which a heterogeneous antiviral CD8^+^ T cells recruited to inflamed airways may undergo functional reprogramming within Type 2‐skewed environments, thereby linking viral infection to amplification of allergic inflammation and disease exacerbation.

### 5.2 Atopic Dermatitis

In atopic dermatitis, Tc2 cells have been identified within both lesional skin and the circulation, where they display a phenotype consistent with tissue homing and retention [79]. Expression of chemokine receptors such as CCR4 and CCR10 supports migration to the skin, where their ligands are produced by keratinocytes and other resident cells [80]. Within the cutaneous environment, Tc2 cells contribute to chronic inflammation through sustained production of Type 2 cytokines, which promote eosinophil recruitment, IgE‐mediated responses and alterations in keratinocyte function [12, 79, 80]. These effects are likely to exacerbate barrier dysfunction and perpetuate inflammatory circuits within the skin. Although Th2 cells remain the dominant drivers of disease, Tc2 cells may provide an additional source of Type 2 cytokines, particularly in chronic or relapsing disease states [81].

### 5.3 Food Allergies

Evidence for Tc2 involvement in food allergy is more limited, with relatively few studies focused on this area and mainly studying CD8^+^ T‐cell subsets found in circulation [82, 83]. Tc2‐like CD8^+^ T cells have been detected within the gut mucosa in murine models, where they may contribute to local Type 2 inflammation [84]. The gastrointestinal tract represents a unique immunological environment, characterised by constant antigen exposure and tightly regulated barrier function [85]. In this context, Tc2‐derived cytokines may influence epithelial integrity and immune cell recruitment. Studies using murine models have suggested that an intraepithelial (IEL) subset of Tc cells can produce IL‐9 upon activation together with IL‐10 and lower granzyme A expression [84]. The development of these cells is dependent on CX3CR1 expressing DCs, suggesting possible cross‐presentation of antigens. Given the expression of PD‐1, it has been shown that these IL‐9^+^ IEL CD8^+^ T cells can suppress CD4^+^ T cells in the intestinal environment [84]. Others have also suggested the presence of a CD4^+^CD8αα^+^ subset with similar immunoregulatory role [86] or a reduction in TGF‐β expressing T cells in gut biopsies taken from allergic children [87]. Thus, suggestive of a potential immunoregulatory role for Tc cells that would warrant further study especially in the context of food allergy.

## 6 Tc2 Cells Versus Th2 Cells: Redundancy or Specialisation?

The identification of Tc2 cells raises important questions regarding their relationship to established Type 2 lymphocyte populations, particularly T helper 2 (Th2) cells. At a functional level, Tc2 and Th2 cells share substantial overlap, as both produce IL‐4, IL‐5 and IL‐13 and contribute to the orchestration of allergic inflammation [4]. This overlap has led to the suggestion that Tc2 cells may represent a redundant source of Type 2 cytokines. However, several features support the notion that Tc2 cells may have specialised roles. Unlike Th2 cells, CD8^+^ T cells recognise antigen presented on major histocompatibility complex Class I molecules, allowing them to respond to distinct antigenic sources, including those derived from intracellular pathways or via cross‐presentation [38, 88]. This difference in antigen specificity may enable Tc2 cells to respond to a distinct range of environmental or tissue‐derived signals. However, the role of innate Type 2 cytokines such as IL‐33 shows consistently an important role in Tc2 effector functions; proving to be more potent inducers than IL‐4 and TCR stimulus alone [13, 18]. Thus, it may be that Tc2 cells are more dependent on these TCR independent cues than their CD4^+^ T helper counterparts. Further studies are needed to define the precise contributions of these distinct activation modes, including analysis of Tc2 TCR repertoires and their predicted epitopes [89].

In addition, CD8^+^ T cells are often characterised by enhanced longevity, the capacity to form long‐lived memory populations, and the ability to establish tissue‐resident compartments at barrier sites [90]. These properties may allow Tc2 cells to persist within inflamed tissues and contribute to chronic or relapsing disease. Their relative resistance to corticosteroids further supports a role in sustaining inflammation despite therapeutic intervention. Taken together, these observations suggest that Tc2 cells are unlikely to be a purely redundant feature of Type 2 immunity. Instead, they may function as amplifiers of Type 2 immunity, reinforcing and sustaining inflammatory responses initiated by other cell types. This model is particularly relevant in chronic allergic diseases, where persistent tissue inflammation may depend on the coordinated activity of multiple lymphocyte populations.

## 7 Therapeutic Implications

In addition to their role in driving eosinophilic inflammation, Tc2 cells have been associated with reduced responsiveness to corticosteroid therapy [91]. In vitro studies suggest that Tc2 cells are relatively refractory to corticosteroids compared with classical CD4^+^ T‐cell populations, raising the possibility that they may persist despite treatment and contribute to ongoing disease activity [13, 92, 93]. This feature may be particularly relevant in patients with severe or steroid‐resistant asthma, where conventional therapies fail to fully suppress Type 2 inflammation [94]. Corticosteroids remain a cornerstone of therapy for many allergic diseases, exerting broad anti‐inflammatory effects through glucocorticoid receptor (GR)–mediated repression of inflammatory gene expression [95]. These agents suppress cytokine production, inhibit immune cell activation, and are highly effective in many patients with Type 2‐driven disease. However, substantial evidence indicates that Tc2 cells are relatively refractory to corticosteroid‐mediated suppression [91]. In vitro studies demonstrate that Tc2 cells retain the capacity to produce IL‐4, IL‐5 and IL‐13 despite corticosteroid exposure, in contrast to CD4^+^ T‐cell populations where cytokine production is more effectively inhibited [91, 92, 93]. Mechanistically, this relative resistance reflects alterations in glucocorticoid receptor signalling, including differences in the balance of GRα and GRβ isoforms, as well as activation of alternative signalling pathways such as p38 MAPK that can impair glucocorticoid responsiveness [96]. In addition, local tissue‐derived factors, including epithelial derived alarmins and lipid mediators, may sustain Tc2 activation in a manner that is less sensitive to steroid‐mediated suppression [13]. The persistence of Tc2 cells under corticosteroid treatment may therefore contribute to ongoing inflammation, particularly in severe or treatment‐refractory disease.

In this context, biologic therapies targeting key mediators of Type 2 inflammation have emerged as effective treatment options. Monoclonal antibodies directed against IL‐5, IL‐4 receptor α (IL‐4Rα) and TSLP have demonstrated significant clinical benefit in diseases such as asthma and atopic dermatitis [97]. Although these therapies were not designed to specifically target Tc2 cells, they are likely to modulate their effector functions through blockade of shared cytokine pathways [98]. For example, inhibition of IL‐5 reduces eosinophilic inflammation that in turn could modulate downstream consequences of Tc2 activity, while IL‐4Rα blockade attenuates broader Type 2 cytokine signalling and Tc2 cell induction [9]. However, these approaches primarily target downstream effector pathways rather than the cellular sources of cytokines. Given the upstream role of TSLP in promoting Type 2 inflammation and enhancing Tc2 cytokine production, blockade with anti‐TSLP therapies such as Tezepelumab may suppress Tc2 activation and effector function, although direct effects on Tc2 cells remain to be defined. Given the prominent role played by IL‐33 in the induction of Tc2 effector cytokines, biologics against this pathway would also be a logical target. However, in clinical trials, there have been inconsistent results with primary outcomes not always met despite a tendency towards a reduction in exacerbation and loss of control events [99]. Whether therapies targeting the IL‐33 axis will ultimately prove successful may depend on improved patient stratification, including factors such as age of disease onset (e.g., Early vs. Late) and the degree of eosinophilic inflammation, as well as selection of clinically relevant endpoints beyond lung function such as exacerbation rates or symptom control [99]. Given the central role of IL‐33 in regulating Tc2 effector function, future studies should determine how IL‐33 blockade influences Tc2 cells relative to other Type 2 lymphocyte populations and whether these effects differ between circulating and tissue‐resident Tc2 cells. Such studies may provide important mechanistic insights into patient responses to IL‐33‐targeted therapies.

Clinical and experimental observations suggest that Type 2 cytokine–producing T‐cell populations, including Tc2 cells, may persist in circulation despite biologic therapy. Consistent with this, treatment with anti–IL‐5 therapies such as mepolizumab effectively reduces eosinophilia and exacerbation frequency but does not necessarily eliminate upstream Type 2 responses [100]. Recent studies further suggest that Tc2 cell populations remain detectable following IL‐5 blockade, supporting the concept that these cells contribute to disease activity and raises the question of whether, if treatment is ceased would these cells re‐enter tissues and restart inflammation in the airways [100]. Given the link between Tc2 frequencies and exacerbation rates in severe asthma [13], it would be interesting if specific targeting of this subset via, for example, bispecific antibodies (e.g., targeting both CD8 and CRTH2 simultaneously), could have greater potential that current therapy options. However, this concept remains to be tested in future studies.

Furthermore, Tc subsets may become increasingly useful as a biomarker for disease severity. For example, GZMK^+^ T cells have been shown to be more robust than traditional Type 2 biomarkers such as eosinophils or tissue IL‐5 expression in a CRS cohort [34]. Although the biomarker potential of Tc2 cells remains to be established, an enrichment of memory CD8^+^ T‐cell populations in overweight/obese asthma and Type 2‐low asthma has been reported, supporting further investigation of these populations as markers of disease heterogeneity [46, 101]. Together, these findings indicate that while current therapies effectively suppress key components of Type 2 inflammation, they may not fully address Tc2‐driven pathways. This limitation may be particularly relevant in chronic or steroid‐resistant disease, where persistent Tc2 activity could contribute to ongoing inflammation and incomplete clinical control.

## Author Contributions

S.d.S.F. and C.A.T. wrote and edited the manuscript.

## Funding

This study was supported by grants from the Swedish Research Council (2020–02170), Swedish Society for Allergology, Konsul Bergh Stiftelse and Center for Innovative Medicine (FoUI‐976197).

## Conflicts of Interest

The authors declare no conflicts of interest.

## Data Availability

Data sharing was not applicable to this article as no datasets were generated or analysed during this study.
